# Changing Epidemiology of Invasive Fungal Disease in Allogeneic Hematopoietic Stem Cell Transplantation

**DOI:** 10.3390/jof7100848

**Published:** 2021-10-10

**Authors:** Pedro Puerta-Alcalde, Carolina Garcia-Vidal

**Affiliations:** Department of Infectious Diseases, Hospital Clinic of Barcelona, C/Villarroel 170, 08036 Barcelona, Spain; cgarciav@clinic.cat

**Keywords:** antifungal, fungal infection, immunosuppression, invasive fungal disease, molds, mortality, pneumonia, prophylaxis, risk factors, yeasts

## Abstract

Invasive fungal disease (IFD) is a common cause of morbidity and mortality in patients with hematologic malignancies, especially among those undergoing allogeneic hematopoietic stem cell transplantation (HSCT). The epidemiology of IFD in HSCT patients has been evolving over the last decades, mainly in relation to changes in HSCT therapies such as antifungal prophylaxis. A progressive decrease in *Candida albicans* infection has been documented, alongside a progressive increase in infections caused by non-*albicans* *Candida* species, filamentous fungi, and/or multidrug-resistant fungi. Currently, the most frequent IFD is invasive aspergillosis. In some parts of the world, especially in north Central Europe, a high percentage of *Aspergillus fumigatus* isolates are azole-resistant. New diagnostic techniques have documented the existence of cryptic *Aspergillus* species with specific characteristics. An increase in mucormycosis and fusariosis diagnoses, as well as diagnoses of other rare fungi, have also been described. IFD epidemiology is likely to continue changing further due to both an increased use of mold-active antifungals and a lengthened survival of patients with HSCT that may result in hosts with weaker immune systems. Improvements in microbiology laboratories and the widespread use of molecular diagnostic tools will facilitate more precise descriptions of current IFD epidemiology. Additionally, rising resistance to antifungal drugs poses a major threat. In this scenario, knowledge of current epidemiology and accurate IFD diagnoses are mandatory in order to establish correct prophylaxis guidelines and appropriate early treatments.

## 1. Introduction

Invasive fungal disease (IFD) is a common cause of morbidity and mortality in patients with hematologic malignancies, especially among those undergoing allogenic hematopoietic stem cell transplants (HSCT). The annual number of HSCT procedures has continuously been rising [1], even in lower income countries, since its inception in the late 1950s. Most importantly, older patients and those with more comorbidities are increasingly undergoing HSCT. This, together with the lengthened survival of these patients, is significantly raising the number of patients susceptible to opportunistic infections.

IFD epidemiology in HSCT is in continuous evolution due to changes in host and transplant characteristics, including antifungal pressure, and diagnostic improvements. The widespread use of antifungal agents as both prophylaxis and treatment, as well as in agriculture, has led to a dramatic increase in antifungal resistance [2]. Improvements in non-invasive diagnostic tests and microbiology laboratories have resulted in a higher likelihood of patients being diagnosed with IFD. Comprehensive knowledge of fungal infection epidemiology in patients undergoing HSCT is essential in order to decide optimal antifungal prophylaxis and initiate empirical antifungal therapy early in patients with suggestive clinical presentations. In this review, we focus on recent literature that concerns epidemiological data on IFD in hematologic HSCT recipients, conducting a comprehensive literature search in the PubMed/MEDLINE database of all English-written articles with the following Mesh terms: (“Stem Cell Transplantation” OR “Hematopoietic Stem Cell Transplantation” OR “Bone Marrow Transplantation”) AND (“Invasive Fungal Infections” OR “Mycoses”).

## 2. IFD Diagnosis and Consensus Criteria

In 2002, a consensus group from the European Organization for Research and Treatment of Cancer/Invasive Fungal Infections Cooperative Group (EORTC) and the Mycoses Study Group (MSG) published standard definitions of IFD for clinical and epidemiological research [3]. These definitions assigned three different levels of probability of IFD (proven, probable, and possible) on the basis of host, clinical, and microbiological criteria. Since then, these definitions have been updated twice, of which one was rather recent, mainly due to growing evidence and advances made in microbiological techniques [4,5].

Culture-based detection remains the gold standard for the diagnosis of IFD. However, proven infections require the specimen to be obtained by a sterile procedure from a normally sterile site (apart from fungemia mostly caused by yeasts). This is often difficult to perform in patients with severe thrombocytopenia. Similarly, histopathological detection of fungi in a sterile sample is also a criterion for proven IFD. In the last update of the EORTC/MSG consensus, amplification of fungal DNA by polymerase chain reaction (PCR) combined with DNA sequencing when fungi are seen in formalin-fixed paraffin-embedded tissue also come to form part of the criteria for proven diagnosis [5].

Another molecular technique that has grown in use is *Aspergillus* PCR. Such an approach has been shown to perform well for screening and diagnosis confirmation of aspergillosis in blood and bronchoalveolar lavage (BAL) fluid [6]. Additionally, *Candida* PCR has good sensitivity and specificity [7], although its use has not become widespread. Regarding candidemia, the US Food and Drug Administration have recent approval of an innovative approach that combines targeted PCR with T2 magnetic resonance. Called T2Candida, it has shown high sensitivity and specificity in detecting Candida species directly in blood specimens [8].

Biomarkers are essential in the current scenario of IFD diagnosis. Serum, BAL, and even cerebrospinal fluid detection of aspergillus galactomannan antigen (GM) are helpful markers in diagnosing invasive aspergillosis [9]. The last EORT/MSG consensus has tried to standardize the diagnostic thresholds for the different specimens [5]. However, performance of GM remains clearly lower in non-neutropenic patients and/or those undergoing mold-active prophylaxis. β-D-glucan (BDG) can be detected in patients with different fungal infections, such as candidiasis, aspergillosis, pneumocystosis or fusariosis, whereas it is absent in zygomycosis. BDG displays an extremely high negative predictive value for these IFD, although a confirmatory positive result is recommended due to its lack of IFD specificity [5,10].

## 3. General Overview of IFD Epidemiology in HSCT and Epidemiological Changes within the Last Decades

### 3.1. The Most Common Fungi Causing IFD in HSCT Recipients

Some of the most important studies detailing the epidemiology of IFD in HSCT are described in Table 1. At the beginning of this millennium, incidence of IFD in different series of HSCT recipients ranged from 10% to as high as 50% [11,12]. In older series, *Candida albicans* was the most common causative agent of IFD (10–25%); associated mortality rates due to candidemia reached 39%, increasing to 90% when tissue invasion occurred [13]. At that time, *Aspergillus* spp. diagnosis occurred in less than 6% of patients, but mortality was almost 100%. Yet, a revolution in antifungal prophylaxis and IFD diagnosis has shifted the epidemiological landscape of IFD in patients undergoing HSCT, with a clear decrease in *Candida* spp. infections and an increase in invasive mold diseases (IMD), mainly caused by *Aspergillus* spp.

Several factors have influenced this epidemiological development, including changes in conditioning regimens, graft-versus-host disease (GVHD) management, and intravascular catheter management. However, the widespread introduction of antifungal prophylaxis is the most likely leading factor in this change in epidemiology.

In the early 1990s, Goodman et al. conducted the first trial comparing prophylactic use of fluconazole vs. placebo in patients undergoing HSCT [14]. Investigators observed that fluconazole use lowered the incidence of systemic and superficial fungal infections and reduced infection-associated mortality. A separate trial performed by Slavin et al. confirmed the findings, with prophylactic use of fluconazole being associated with improved 110-day survival [15]. Both trials observed an incidence of fungal infection of 16% in the placebo arm, with an approximate 90% of IFD caused by *Candida* spp. Finally, another trial by Marr et al. demonstrated an association between fluconazole use and protection against *Candida* infections, alongside reduced GVHD and improved overall survival [16].

Following these trials, antifungal prophylaxis with fluconazole in HSCT recipients became the standard of care in most centers around the world and is a recommendation set by most international guidelines [17]. Lately, posaconazole has demonstrated superiority to fluconazole in preventing invasive aspergillosis and reducing mortality related to fungal infections in patients with GVHD [18]. For this reason, most centers have established its use for this indication too.

A few prospective studies have evaluated the composition of IFD in HSCT after the vast introduction of antifungal prophylaxis. In 2010, the Transplant-Associated Infections Surveillance Net (TRANSNET) reported 2001–2006 IFD epidemiology in HSCT recipients across a network of 23 transplant centers in the United States [19]. In this study, Kontoyiannis et al. documented a proven or probable IFD in 9.2% of allogenic HSCT recipients. Invasive aspergillosis was the most common IFD, causing 43% of cases, followed by invasive candidiasis (28%) and zygomycosis (8%).

The Prospective Antifungal Therapy (PATH) Alliance registry documented proven or probable IFD in HSCT recipients across 16 medical centers from North America between 2004 and 2007 [20]. Approximately 60% of IFD cases were due to *Aspergillus* spp., while 25% of IFD cases were due to candidiasis and 7% due to both zygomycosis and other molds. Investigators did not report rates of prior antifungal treatment in either the TRANSNET or PATH cohorts.

The Italian HSCT Cooperative Group (GITMO) evaluated 1858 patients undergoing an allogenic HSCT across 30 transplant centers between 2008 and 2010 [23]. In that study, 95% of patients received antifungal prophylaxis (75% primary fluconazole prophylaxis; 15% primary mold-active prophylaxis; and 5% secondary prophylaxis), and 1 year cumulative incidence of proven or probable IFD was 8.8%. Once again, invasive aspergillosis was the most common infection (81.1%), followed by invasive candidiasis (11.0%), zygomycosis (3.7%), and fusariosis (1.8%).

Nucci et al. reported data on a prospective cohort of eight transplant centers in Brazil between 2007 and 2009 [22]. In this cohort, 90% of allogenic HSCT recipients received antifungal prophylaxis, primarily with fluconazole (91%). One year cumulative incidence of IFD was 11.3%. Remarkably, the leading IFD was fusariosis (35%), followed by aspergillosis (30%), invasive candidiasis (17%), and hyalohyphomycosis (12%). These results highlight the importance of geographical and environmental context in fungal epidemiology and antifungal susceptibility. However, it should be noted that galactomannan testing was not routinely performed in this study, thereby possibly contributing to an underestimation of the real incidence of aspergillosis. A rather recent, prospective study by the same group (2015–2016) showed that invasive aspergillosis was, indeed, the most frequent IFD (56%), followed by candidemia (24%) and fusariosis (12%) [27].

Finally, one of the last prospective studies in this setting was that done across 31 HSCT centers in China [24]. Of the total number of allogenic HCST patients, 86% received prophylaxis (61%, fluconazole; 22%, itraconazole; and 19%, voriconazole). Despite these high rates of anti-mold prophylaxis, 6 month cumulative incidence of IFD was 9.2%. In this study, *Aspergillus* spp. and *Candida* caused 71% and 28% of identified cases, respectively.

### 3.2. Time since HSCT to IFD

Three different periods have been typically defined in the post-HSCT setting: (i) pre-engraftment; (ii) early post-engraftment; and (iii) late post-engraftment. The time interval between HSCT and the IFD appears to have increased, with a shift occurring from early- to late-onset infections. This may be attributable to the rising use of peripheral blood stem cells and/or reduced intensity conditioning procedures, which are often associated with a shorter duration of neutropenia and, thereby, lower risk of fungal infection in the early period. However, length of post-transplant periods is not well standardized; some variability exists among the different studies. In the Italian transplant cohort [23], 57% of IFD diagnoses took place during the early period (defined as within 40 days of HSCT); 24% during early post-engraftment (41 to 100 days after HSCT); and 19% during late post-engraftment (>100 days from transplant). In the TRANSNET cohort, median time after HSCT to IFD was 61 days for candidiasis, 99 days for aspergillosis, 123 days for fusariosis, and 135 days for zygomycosis [19]. Specifically, in the case of invasive aspergillosis, “only” 22% of episodes occurred within the first month, while almost half of all total episodes were diagnosed in the four-month period following HSCT. The PATH registry obtained similar results, with median time from transplant to IFD being 83 days for invasive aspergillosis and 108 days for invasive candidiasis [20]. IFI due to zygomycetes and other molds occurred later after HSCT, with a median time of 162 days since HSCT.

Although neutropenia has been classically identified as the main risk factor for IFD, the aforementioned shift to later IFD underlines the importance and relevance of other risk factors, such as GVHD and its associated severe immunosuppressive treatment, viral infections, lymphopenia, and cellular immunity dysfunction in IFD risk. However, both risk and timing of IFD after HSCT are highly conditioned by the length of antifungal prophylaxis and the drug used; variability among centers would be, in turn, high.

### 3.3. IFD Epidemiology in Pediatric HSCT Recipients

As with adults, improvements in medical care have resulted in an increased burden of IFD risk in children undergoing HSCT. Yet, despite the fact that mortality rates have been reported as high as 50–60%, limited data is available regarding epidemiology, treatment, and overall management of IFD in this population [25,26,28].

IFD incidence rates of approximately 7–16% have been reported in pediatric allogenic HSCT recipients. Most IFD cases (around 50%) occur during pre-engraftment [29], although some series have reported a predominance of cases during the late post-engraftment period [26]. Unlike in adult patients, yeasts and *Candida* spp. particularly remain the most frequent causative agents of IFD in pediatric HSCT recipients [25,26,28]. The second most common causative fungus is *Aspergillus* spp., which is responsible for most IMD. The epidemiology of IFD caused by other yeasts or molds in this population has not been well defined [29,30].

## 4. Risk Factors for IFD in HSCT Recipients

Risk factors for yeast and mold infections are significantly different in general. How-ever, some common risk factors, such as those related to the host, transplants or concomitant infections, influence the risk of IFD.

### 4.1. Host Issues

Older age has been linked with an increased risk of IFD. Diabetes mellitus and hyperglycemia impair innate immunity and have been particularly associated with an elevated risk of mucormycosis. Some prognostic scores in HSCT patients have been shown to correlate with the risk of IFD [31].

Glucocorticoids are a common risk factor for IFD due to neutrophil chemotaxis and oxidative burst inhibition, as well as hindrance of macrophages’ capacity to remove conidia [32]. Delayed engraftment and prolonged neutropenia continue to be some main risk factors for IFD and are associated with treatment failure and increased mortality [33,34].

The main risk factors for disseminated yeast infection (mainly candidemia) are as follows: (i) disruption of the cutaneous barrier, mostly due to intravascular access devices (which may lead to catheter-related fungemia); (ii) disruption of the mucosal barrier due to gastrointestinal tract surgery or GVHD; (iii) alteration of normal bacterial flora due to broad-spectrum antibiotics (leading to yeast overgrowth and predominance); and (iv) total parenteral nutrition (with lipid formulations favoring fungal invasion).

### 4.2. Transplant Issues

Active or refractory disease at the time of the transplant is a major risk determinant. Graft source and donor relatedness have an impact on transplant-related toxicity and the risk of IFD [35]. Compared to peripheral blood stem cells, bone marrow and umbilical cord transplants are associated with the highest risk of IFD due to delayed immune reconstitution. Similarly, haploidentical transplants and, to a lesser extent, stem cell donors who are not matched siblings are associated with longer immunosuppression and an increased risk of IFD [21,36]. GVHD poses an extremely high risk of IFD due to both its own immunosuppressive effect, and the required immunosuppressive treatment that often includes high-dose steroids [37].

### 4.3. Concomitant Infections

Cytomegalovirus is associated with an increased risk of IFD in the post-transplant setting. This association is perhaps due to the virus’ deleterious effect on innate immunity. Respiratory viruses, such as the influenza virus, respiratory syncytial virus, adenovirus, and parainfluenza, impair mucociliary activity, local innate immunity and systemic host defenses, and are thus associated with a higher risk of IFD [38,39]. This association is particularly relevant in patients with severe influenza and severe SARS-CoV-2 who require ICU admission [40,41].

## 5. The Most Common Causative Agents

### 5.1. Aspergillus *spp.*

*Aspergillus* spp. are ubiquitous, environmental molds, forming spores that enter the body via respiratory inhalation. As already mentioned, invasive aspergillosis is currently the most common IFD in HSCT recipients, mainly with pulmonary involvement. *Aspergillus fumigatus* is the most common causative species, probably due to the relatively small size that characterizes *A. fumigatus* conidia, which allows for its deep penetration into the alveolar space. Additionally, *A. fumigatus* can grow in high temperatures (37–50 °C), being more resistant and thermotolerant than other *Aspergillus* species [39]. In the TRANSNET and PATH studies, 44% and 37% of aspergillosis cases, respectively, were due to *A. fumigatus*, although in a considerable percentage of cases, identification at the species level was not achieved [19,20]. However, invasive aspergillosis caused by non-*fumigatus* species seems to be increasing. In a study performed at the Fred Hutchinson Cancer Research Center comparing two close periods (1993–1995 vs. 1996–1998), invasive aspergillosis caused by non-*fumigatus* species rose from 18% to 34% [42]. Nonetheless, these changes could just reflect improvements made in microbiological identification, which may increase with the advent of molecular techniques. Additionally, this seems to be dependent on whether or not it is a breakthrough episode [43]. The most commonly isolated non-*fumigatus* species are *Aspergillus niger*, *Aspergillus terreus*, and *Aspergillus flavus*.

Antifungal resistance in *Aspergillus* is a huge threat. Since the beginning of the century, different studies have reported an increasing prevalence of azole resistance in *A. fumigatus* [44,45]. Interestingly, this antifungal resistance is widely mediated by mutations in a specific gene (TR34/L98H) and could have occurred due to vast use of triazole fungicides in agriculture [2]. Besides this mutation, some *Aspergillus* species are known to have varying susceptibilities to different antifungal drugs [46]. For example, *Aspergillus terreus* is typically less susceptible to amphotericin B. Additionally, the use of molecular tools has permitted the description of new cryptic species among different *Aspergillus* species complexes. Remarkably, these species commonly exhibit innate, high-level resistance to multiple antifungal agents, including amphotericin B and the triazoles [47,48]. This observation underlines the importance of identifying to the species level and performing antifungal resistance testing, especially in those cases wherein a lack of response to initial therapy is present.

### 5.2. Candida *spp.*

*Candida* spp. forms part of the gastrointestinal tract and skin microbiota. Loss of mucosal and skin barrier integrity—as occurs in mucositis, GVHD, and endovascular catheters—may lead to invasive candidiasis in HSCT recipients. Invasive candidiasis is the second most common cause of IFD in HSCT recipients.

*Candida albicans* had classically been the most common IFD-causing pathogen. However, the widespread use of azole prophylaxis not only decreased the overall incidence of invasive candidiasis, but also increased the percentage of cases caused by non-*albicans* species. In the PATH cohort, 76% of invasive candidiasis episodes were caused by non-*albicans* species, with 44% and 11% of episodes caused by *Nakaseomyces glabrata* (formerly known as *Candida glabrata)* and *Pichia kudriavzevii* (formerly known as *Candida krusei*), respectively [20]. Similar results have been reported in other series [12,19]. Current candidiasis epidemiology is mainly determined by antifungal selection pressure [49,50]. This should be considered when invasive candidiasis is clinically suspected or diagnosed. In such a challenging scenario, implementing a bundle of measures, including initial treatment (adequate antifungal and source control within 72 h), identification of complicated candidemia (follow-up blood cultures, ophthalmoscopic evaluation, and echocardiography in at-risk patients), and final treatment adequacy (de-escalation when indicated and adequate length of antifungal treatment), has proven to reduce mortality [51].

### 5.3. Zygomycetes

Mucormycosis-producing agents are ubiquitous fungi, commonly found in decaying organic matter that cause infection in patients with hematologic malignancies and HSCT recipients, and are characterized by violent evolution with frequent angioinvasion, tissue infarction, and necrosis [52,53]. In different prospective cohorts of HSCT recipients with IFD, mucormycosis accounted for approximately 4–8% of cases [19,20,23]. By contrast, in the Chinese cohort, there was only a single case of mucormycosis diagnosed among the 1401 transplanted patients [24]. Potential explanations for these differences include environmental factors and factors related to the use of different diagnostic approaches.

Some groups from various countries have reported an increasing incidence in mucormycosis in patients with hematologic malignancies [54,55]. This rise in mucormycosis incidence could be partially due to selection pressure by voriconazole use. It could also be due to increased long-term immunosuppression of HSCT patients and decreased aspergillosis-related mortality, which results in the eventual emergence of rarer molds later after transplantation [20]. Furthermore, no specific antigenic diagnostic methods for Zygomycetes exist, and histological findings serve as the basis for most diagnoses. Therefore, mucormycosis cases may be assumed as being underdiagnosed currently.

### 5.4. Hyalohyphomycoses

Hyalohyphomycoses is a term referring to IFD caused by non-pigmented molds (other than the genera *Aspergillus* or *Penicillium* or the class *Zygomycetes*) that form hyphal elements with hyaline or clear walls in tissue. Most representative molds in these group include *Fusarium*, *Scedosporium*, *Lomentospora*, *Acremonium*, and *Paecilomyces* spp. Although IFD caused by hyalohyphomycetes are very uncommon, exceedingly high mortality rates of up to 90% have been reported [56,57]. The most frequently isolated mold in this group is *Fusarium* spp., accounting for 3% and 7% of all IFD in the TRANSNET and PATH studies, respectively [19,20]. However, hyalohyphomycoses are probably highly influenced by both the host’s degree of immunosuppression and the geographical context. In this regard, fusariosis represented 12–35% of all IFD cases in Brazilian cohorts [22,27].

### 5.5. Pneumocystis jirovecii

*Pneumocystis jirovecii,* which was long thought to be a protozoan organism, is an ascomycetous fungi that classically causes pneumonia in immunosuppressed patients. Before the introduction of antibiotic prophylaxis, the risk of *Pneumocystis* pneumonia was around 5–15% in patients receiving a HSCT [58,59]. This has dramatically decreased since the widespread implementation of prophylactic strategies initiated in the 1980s; *Pneumocystis jirovecii* pneumonia represented less than 1.5% of all IFD in the current HSCT cohort studies [19,20,23]. Indeed, recent cohorts have shown that most pneumocystosis cases in this population occur late after HSCT in patients who were no longer receiving *Pneumocystis* prophylaxis [60,61].

### 5.6. Other

*Cryptococci* are basidiomycetous, encapsulated yeasts that typically cause disseminated and/or central nervous system infections in immunocompromised patients, e.g., *Cryptococcus neoformans* or *C. gattii.* Delayed reconstitution of CD4^+^ lymphocytes and B lymphocytes after allogenic HSCT places these patients at a theoretically high risk of cryptococcal infection. However, prevalence of cryptococcosis in HSCT is very low (<1%), probably due to effective antifungal prophylaxis [19,62].

Non-*Candida* opportunistic yeasts are emerging causes of bloodstream infections in immunosuppressed patients with hematologic malignancies. However, scarce information is available regarding this type of IFD in HSCT recipients. In a retrospective study conducted at the MD Anderson Cancer Center, the most commonly rare (non-*Candida*, non-*Cryptococcus*) yeasts to cause bloodstream infections in patients with cancer were *Rhodotorula, Trichosporon, Saccharomyces*, and *Geotrichum* [63]. However, in this study, only 29% of the patients had received a prior HSCT.

## 6. Breakthrough IFD: The New Reality of IFD in HSCT Recipients

Current IFD epidemiology in HSCT recipients is highly conditioned by the widespread introduction of antifungal prophylaxis. In this setting, there is an increasing number of centers using mold-active antifungals (mainly mold-active triazoles and/or echinocandins) as primary prophylaxis. Further, a prior IFD is no longer a contraindication of HSCT, and the number of patients receiving secondary prophylaxis (or treatment) for prior infections is on the rise [35]. In this context, breakthrough IFD is therefore likely associated with a further epidemiological change and poses a great challenge to treating physicians [64]. For example, in a retrospective study including HSCT recipients, Lamoth et al. described the epidemiology of IMD episodes occurring in a certain period, considering such episodes as breakthrough or not. An increased number of mucormycosis cases (from 15% to 31%) was reported in breakthrough IMD episodes to voriconazole or posaconazole.

Prior antifungal treatment highly influences breakthrough IFD epidemiology.

### 6.1. Breakthrough Infections to Posaconazole

Posaconazole is the most widely used mold-active azole, showing good activity against *Aspergillus* as well as against Zygomycetes. As previously stated, it has been incorporated in prophylaxis in patients receiving a HSCT. In the most important prophylaxis trial [18], 5.3% of patients in the posaconazole group presented a breakthrough IFD, of which 44% were caused by *Aspergillus* spp., 25% by *Candida* spp., and 31% by other molds. Some unicentric and retrospective studies later reported varying incidences ranging between 3% and 11% [65,66]. In these cohorts, rates of invasive aspergillosis among diagnosed IFD were relatively low, with a high proportion of breakthrough episodes caused by Zygomycetes and rare yeasts. However, at that time, posaconazole efficacy was limited by erratic absorption of posaconazole solution [67]. Data on breakthrough episodes to posaconazole tablets in HSCT is very scarce. A retrospective unicenter study evaluated over three hundred patients with hematologic malignancies receiving posaconazole prophylaxis, of whom 70 had undergone a HSCT [68]. The overall rate of breakthrough IFD was 2%, increasing to 4% (3 of 70) when considering HSCT recipients. These three cases were one aspergillosis, one rare mold (*Penicillium*), and one rare, unidentified yeasts infection. All three patients died within six weeks.

### 6.2. Breakthrough Infections to Voriconazole

Two randomized control trials have evaluated the use of voriconazole prophylaxis in the HSCT setting [69,70]. Wingard et al. reported a 1 year cumulative incidence of IFD of 13% (including proven, probable, and presumptive IFD) in the voriconazole arm. The most common IFD was aspergillosis (41%), but *Aspergillus* species were not identified [69]. In the second trial, Marks et al. only documented three breakthrough IFD episodes to voriconazole (one *A. fumigatus*, one *P. kudriavzevii*, and one *C. parapsilosis*). However, significantly different information has been documented in “real-life” retrospective cohort studies [71,72,73,74]. Most of these studies report breakthrough infection incidence of around 2–7%, with most episodes caused by Zygomycetes, remarkably. Candidemia due to *P. kudriavzevii* and *N. glabrata* were also frequent. These data seem expectable due to reduced susceptibility of these pathogens to voriconazole, even though it demonstrates excellent activity against *A. fumigatus.*

Voriconazole level has large interpersonal variability, given that it depends on several factors, i.e., the patient’s age, potential drug–drug interactions, and cytochrome P450 polymorphism [75]. In this setting, therapeutic drug monitoring is mandatory whenever there is clinical suspicion of breakthrough IFD.

### 6.3. Breakthrough Infections to Echinocandins and Other Antifungals

Four randomized clinical trials have evaluated echinocandins versus azoles as prophylaxis in patients receiving a HSCT, showing contradictory results [76,77,78,79]. Incidence of breakthrough infections in echinocandins ranged between 2–7%. Though some of these studies did not report on isolated fungi, invasive aspergillosis and invasive candidiasis seemed to be the most common entities. Some cohort studies on HSCT recipients presenting breakthrough episodes to echinocandins have reported similar incidence rates [80,81,82,83]. Remarkably, despite good in vitro activity by echinocandins against *Aspergillus* spp., most breakthrough episodes in these studies were invasive aspergillosis.

To our best knowledge, data are missing concerning breakthrough episodes to isavuconazole or amphotericin B in HSCT patients.

### 6.4. A Recommended Approach to HSCT Recipients with Suspicion of Breakthrough IFD

Breakthrough fungal infection is associated with an exceedingly high mortality [64]. In this context, clinicians should perform an early and aggressive diagnostic work-up in those patients with clinically suspicion of having a breakthrough IFD. Early CT imaging +/− bronchoscopy with BAL performance should take place. In cases wherein BAL results are negative, clinicians should perform a transthoracic or transbronchial biopsy when feasible. Additionally, whenever fungal isolation occurs, identification to the species level and assessment of antifungal susceptibility are highly recommended.

**Figure 1:**
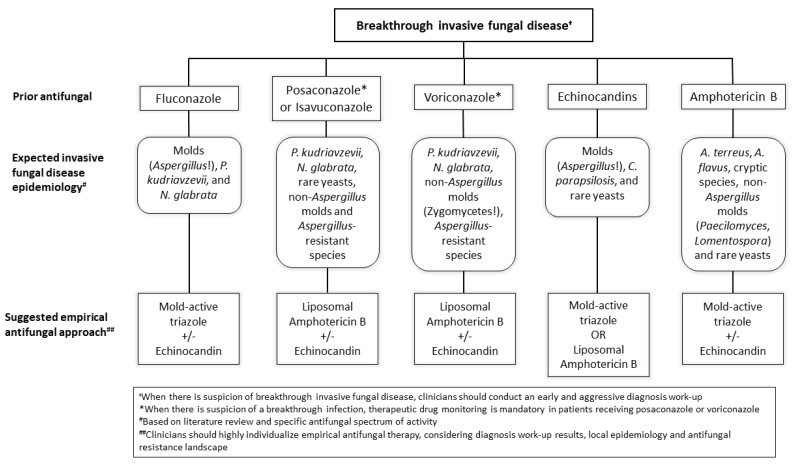
Algorithm of expected breakthrough invasive fungal disease epidemiology per prior antifungal therapy and suggested empirical treatment.

As previously mentioned, breakthrough IFD epidemiology is mainly determined by prior antifungal therapy. Figure 1 summarizes the “expected” epidemiology per prior antifungal therapy, as well as our personal recommendations on potentially empirical treatment in cases of suspected breakthrough IFD.

## 7. Conclusions and Future Perspectives

IFD epidemiology in HSCT recipients has been changing in recent decades, mainly following the widespread use of antifungal prophylaxis. Currently, the most frequent IFD is invasive aspergillosis, mainly due to *Aspergillus fumigatus*. The second most common IFD is invasive candidiasis, with most current cases caused by non-*albicans* species. Mucormycosis and fusariosis follow thereafter, considering frequency, and are associated with very high mortality. IFD caused by rarer molds and yeasts are uncommon but appear to be increasing in patients undergoing a HSCT.

In the coming years, IFD epidemiology is likely to keep changing due to an increased use of mold-active antifungals. At the same time, improvements in microbiology laboratories and techniques, as well as the generalization of molecular diagnoses will help characterize the real epidemiology of fungal infections more precisely in these patients. For a nuanced description, it is important to identify to the species level; it could have an impact on antifungal resistance. Finally, increasing antifungal resistance in *Aspergillus*, but also in overall IFD, poses a major threat. In this scenario, knowledge of current epidemiology and accurate diagnosis of IFD remain crucial to establishing correct prophylaxis guidelines and appropriate early treatments.

## Figures and Tables

**Table 1:** Important studies regarding invasive fungal disease in allogenic HSCT recipients.

Reference and Year of Publication ^&^	Study Type and Period	n	Prophylaxis	IFD Incidence	IFD Epidemiology	Time from HSCT to IFD
Martino et al. [11] 2002	Retrospective study 1996–2000	395 allo-HSCT	73% fluconazole, 17% itraconazole, 4% amphotericin B, 6% no prophylaxis	14%	64% aspergillosis, 20% candidiasis, 6% mucormycosis, 6% other	Median day post HSCT: +90 days (range +4 to +522) Post-HSCT periods: 19% <21 days, 32% 21–90 days, 49% >90 days
Pagano et al. [12] 2007	Retrospective study 1999–2003	1249 allo-HSCT	39% fluconazole, 21% itraconazole	8%	81% aspergillosis, 14% candidiasis (50% non-*albicans*), 3% fusariosis, 2% other molds	50% yeast infections and 36% mold disease occurring >100 days post HSCT
Garcia-Vidal et al. [21] 2008	Retrospective study 1998–2002	1248 allo-HSCT	Not reported	13% invasive mold disease	87% aspergillosis, 4% fusariosis, 3% mucormycosis	Post-HSCT periods: 22% <40 days, 40% 40–100 days, 38% >100 days
Neofytos et al. [20] 2009	Prospective study 2004–2007	161 IFD in allo-HSCT	Not reported	Not applicable	57% aspergillosis, 25% candidiasis, 7% mucormycosis, 8% other molds	Median days post HSCT (range): 83 days (3–6542) for aspergillosis, 108 days (0–2219) for candidiasis, 162 days (7–932) for mucormycosis and other mold diseases
Kontoyiannis et al. [19] 2010	Prospective study 2001–2005	6666 allo-HSCT	Not reported	≈8%	43% aspergillosis, 28% candidiasis, 8% mucormycosis, 10% other molds	Median days post HSCT: candidiasis, 61 days; aspergillosis, 99 days; fusariosis, 123 days; mucormycosis, 135 days
Nucci et al. [22] 2013	Prospective study 2007–2009	378 allo-HSCT	81% fluconazole, 1% itraconazole, 4% voriconazole, 4% amphotericin B	11%	35% fusariosis, 30% aspergillosis, 17% invasive candidiasis, and 12% hyalohyphomicosis	Median (IQR) days post HSCT: 53 (19–232) days
Girmenia et al. [23] 2014	Prospective study 2008–2010	1858 allo-HSCT	75% fluconazole, 14% mold-active prophylaxis (NS), 5% secondary prophylaxis (NS), 6% no prophylaxis	9%	81% aspergillosis, 11% candidiasis, 4% mucormycosis, 2% fusariosis, 1% other molds, 1% rare yeasts	Post-HSCT periods: 57% <40 days, 24% 40–100 days, 19% >100 days
Sun et al. [24] 2015	Prospective study 2011	1053 allo-HSCT	61% fluconazole, 22% itraconazole, 19% voriconazole	9%	33% aspergillosis, 13% candidiasis, 54% non-identified	Median (IQR) days post HSCT: 45 (16–93) days
Gomez et al. [25] 2018	Retrospective study Pediatric patients 1998–2016	143 allo-HSCT	Fluconazole or voriconazole (rates not reported)	13%	86% candidiasis, 17% aspergillosis	Not reported
Linke et al. [26] 2019	Retrospective study Pediatric patients 2005–2015	221 allo-HSCT	52% fluconazole, 9% mold-active azole, 32% liposomal amphotericin B, 1% micafungin, 6% no prophylaxis	7%	73% aspergillosis, 27% candidiasis	Post-HSCT periods: 33% pre-engraftment, 13% engraftment-180 days, 53% >180 days
Souza et al. [27] 2020	Prospective study 2015–2016	71 allo-HSCT	68% fluconazole, 17% micafungin, 11% mold-active azole (NS)	11%	50% aspergillosis, 38% candidiasis, 12% other molds	Not reported

^&^ Arranged chronologically. Abbreviations. IFD: invasive fungal disease; HSCT: hematopoietic stem cell transplantation; allo-HSCT: allogenic HSCT; IQR: interquartile range; NS: not specified.

## Data Availability

Not applicable.
